# Addressing the Immunogenicity of the Cargo and of the Targeting Antibodies with a Focus on Deimmunized Bacterial Toxins and on Antibody-Targeted Human Effector Proteins

**DOI:** 10.3390/biomedicines5020028

**Published:** 2017-06-02

**Authors:** Yehudit Grinberg, Itai Benhar

**Affiliations:** Department of Molecular Microbiology and Biotechnology, The George S. Wise Faculty of Life Sciences, Tel-Aviv University, Tel-Aviv 69978, Israel; z_yehudit@hotmail.com

**Keywords:** deimmunization, immunotoxins, immunocytokines

## Abstract

Third-generation immunotoxins are composed of a human, or humanized, targeting moiety, usually a monoclonal antibody or an antibody fragment, and a non-human effector molecule. Due to the non-human origin of the cytotoxic domain, these molecules stimulate potent anti-drug immune responses, which limit treatment options. Efforts are made to deimmunize such immunotoxins or to combine treatment with immunosuppression. An alternative approach is using the so-called “human cytotoxic fusion proteins”, in which antibodies are used to target human effector proteins. Here, we present three relevant approaches for reducing the immunogenicity of antibody-targeted protein therapeutics: (1) reducing the immunogenicity of the bacterial toxin, (2) fusing human cytokines to antibodies to generate immunocytokines and (3) addressing the immunogenicity of the targeting antibodies.

## 1. Therapeutic Monoclonal Antibodies

**Figure 1:**
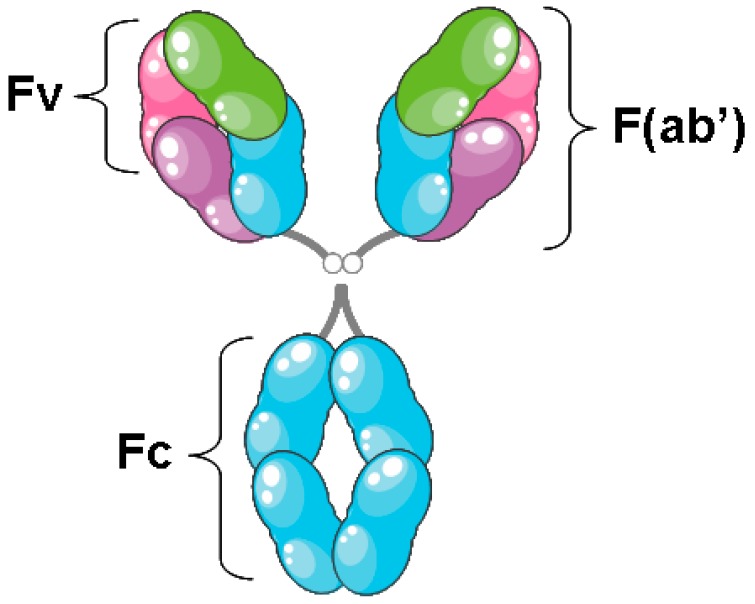
Schematic structure of an antibody. Pink, the variable domain of the light chain (VL); purple, the constant domain of the light chain (CL); green, the variable domain of the heavy chain (VH); blue, the constant domain of the heavy chain (CH_1–3_). The “fragment variable” (Fv) comprises the VH and VL domains of an antibody held together by non-covalent association. The Fv is the most important region for binding to antigens. The “fragment antigen-binding” (F(ab’)) fragment is a region on an antibody that binds to antigens, comprising of one constant and one variable domain of each of the heavy and the light chains. The “fragment crystallizable” region (Fc) is the tail region of an antibody that interacts with cell surface receptors called Fc receptors and some proteins of the complement system. The Fc region is composed of two identical protein fragments, derived from the second (CH_2_) and third (CH_3_) constant domains of the two antibody heavy chains.

Antibodies are homodimers consisting of two heavy chains and two light chains. Each light chain consists of one variable domain (VL) and one constant domain (CL), and each heavy chain consists of one variable domain (VH) and three constant domains (CH_1_, CH_2_, CH_3_) [1] (Figure 1).

The VH and VL domains compose the antigen-binding domain of the Antibody, termed **Fv**. The fragment antigen-binding (Fab) is composed of the Fv domain along with the CL and CH_1_ regions. The Fc domain is composed of the CH_2_ and CH_3_ and is responsible for the antibody’s effector abilities.

**Figure 2:**
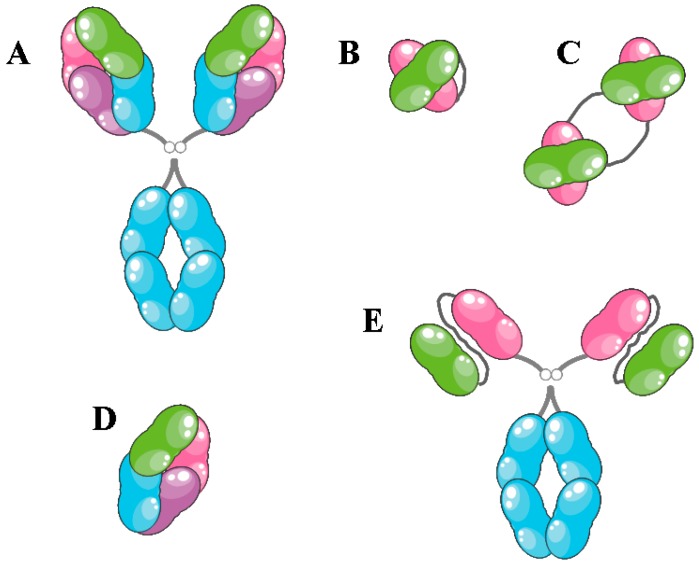
Schematic representation of different antibody fragments. (**A**) Full IgG, ~150 kDa; (**B**) single chain Fv (scFv), the variable domain of each chain connected by a linker, ~25 kDa; (**C**) diabody, two scFvs connected by two linkers, ~50 kDa; (**D**) F(ab’) domain, ~50 kDa; (**E**) scFv–Fc fusion, ~100 kDa. The color scheme is identical to that of Figure 1.

Antibody molecules and their derivatives have a great potential for use in a variety of research, diagnostic and therapeutic applications. Specifically, monoclonal antibodies (mAbs) are extremely useful molecules for biotechnological and biomedical applications due to their high affinity and specificity. Most of the therapeutic mAbs are of the bivalent immunoglobulim G (IgG) isotype and can therefore bind two identical antigens. This avidity effect can enhance the apparent affinity of the antibody. The Fc part of the heavy chain is involved in non-antigen-binding functions (effector functions) of the antibody, such as antibody-dependent cellular cytotoxicity (ADCC), complement dependent cytotoxicity (CDC) or antibody-dependent phagocytosis (ADCP) [2]. In addition to full-size IgGs, smaller antibody fragments can be used to create novel molecules with a specific subset of functional properties (Figure 2) [3].

Therapeutically-used antibodies can be classified into two categories. The first, to which most approved therapeutic antibodies belong, are “un-armed” antibodies, in which the antibody binds the target only (such as a receptor antagonist) to recruit effector mechanisms. The second, “armed antibodies”, are antibodies acting as “guided missiles” that carry a cargo and deliver it to the target cells [4]. Delivery of pharmaceuticals by antibodies or their fragments requires specific antigens that are differentially expressed or over-expressed at the site of tumor or disease and are undetectable in healthy tissues.

## 2. Recombinant Immunotoxins

### 2.1. Background

Immunotoxins (ITs), designed to treat cancer, are hybrid proteins comprised of a targeting moiety, mostly single chain Fv (scFv) or Fab antibody fragments, fused or chemically conjugated to a cytotoxic moiety. ITs can generally be divided into three generations: (i) first generation ITs were produced by chemically conjugating native toxins to whole murine antibodies; these ITs had some drawbacks, like lack of specificity, low stability and heterogeneous composition; (ii) second generation ITs were fabricated by chemically conjugating a toxin fragment with no targeting activity following removal of the autonomic cell-binding domains to the murine antibody; this method yielded increased amounts of IT molecules that could be safely administered to animals and humans, but heterogeneity issues persisted; (iii) third generation ITs are produced by recombinant DNA techniques; these ITs, designated recombinant immunotoxins (RITs), mostly consist of an scFv fragment fused to the truncated toxin fragment [5,6,7,8].

The cytotoxic moiety can be of plant (e.g., ricin from *Ricinus communis*) or bacterial (e.g., *Diphtheria* toxin (DT) or *Pseudomonas* exotoxin A (PE)) origin [9]. After cellular internalization, these highly potent toxins induce cellular cytotoxicity via inhibition of protein synthesis. In addition, they are active in very small amounts. It has been shown, for example, that cell death can be induced by a single DT molecule in the cytoplasm [10]. Most of the RITs currently under clinical evaluation consist of either DT or PE, which are easily produced as recombinant proteins in *Escherichia coli* and have been shown to be more active and cause fewer side effects in humans [7]. These properties, as well as their extensively studied mechanism of action, make them good candidates for this purpose.

### 2.2. Toxicity

When a non-human protein-based drug is injected into human patients, an immune response is very likely to arise during treatment. This response involves anti-drug antibodies (ADAs) and also, in some cases, T-cells, as well, and may neutralize the clinical effect of the drug [11]. In addition, serious adverse effects, such as infusion or allergic reactions, anaphylaxis, delayed hyper sensitivity and autoimmunity, can be associated with the formation of neutralizing Abs (NAbs) against the foreign protein [12].

Since first and second generation ITs were constructed using murine antibodies, human anti-mouse antibodies (HAMA) were formed upon their administration to patients, thus limiting further IT treatment. To address this issue, antibodies were humanized and/or their fragments were used [13,14,15]. While antibody chimerization and humanization were very significant for the development of therapeutic mAbs, they were of little consequence for the development of ITs, where most of the ADA and T-cell responses result from epitopes of the non-human toxic protein.

As mentioned above, the toxins used for the construction of RITs are of non-human origin and are thus highly immunogenic. This restricts the number of doses each patient can tolerate to only one or a few. Over the past 16 years, extensive effort and resources were dedicated to reducing the immunogenicity of such RITs [16,17,18,19]. This section will focus on reducing the immunogenicity of the PE component of PE-based ITs.

### 2.3. Pseudomonas Exotoxin A

*Pseudomonas* exotoxin A (PE) is one of the most commonly-used toxins in the construction of RITs for targeted cancer therapy. PE is composed of three structural and functional domains: Domain Ia, which is responsible for target-cell recognition, Domain II, which mediates translocation across the cell membrane, and Domain III, which is responsible for the adenosine-di-phosphate (ADP)-ribosylation of elongation factor 2 (EF2), causing the arrest of protein synthesis and eventually apoptotic cell death [13]. The reasons for the extensive use of PE in the construction of RIT are that this is one of the most studied toxins, with a very well-documented killing activity and mechanism of action [20,21]. In addition, PE and PE-based protein domains can be easily produced in *Escherichia coli* (*E. coli*) expression systems. Furthermore, PE can withstand many mutations without compromising its activity. This feature enabled increasing PE stability and significantly reducing its immunogenicity, making it an even better candidate for cancer treatment [8,13,18,21,22,23,24].

The most common form of PE used for making RITs is a 38-kDa fragment designated PE38 [25]. PE38 was generated by the deletion of a large portion of domain Ia and a small portion of domain Ib [24]. PE-based ITs have been studied since the mid-1980s, with most of the studies described herein carried out by the group of Ira Pastan at the US National Institutes of Health (NIH).

### 2.4. PEGylation

One approach to reduce non-specific toxicity is to covalently attach polyethylene glycol (PEG) chains to the therapeutic protein [26,27]. Such attachment has been found to be helpful in masking conformational immunogenic epitopes of the protein from the immune system. However, PEGylation of the protein increases its size, thus affecting its serum half-life and clearance rate, which in turn affects its pharmacokinetics. PEGylation of RITs was already performed in the 1990s [23,28,29]. The addition of PEG markedly reduced the cytotoxic activity of the RIT [30], thus precluding this approach.

### 2.5. B-Cell Epitope Identification and Elimination

B-cell epitopes are generally located at a small number of particular sites on the surface of the protein. The major group of neutralizing B-cell epitopes is discontinuous conformational epitopes [31]. Attempts to identify B-cell epitopes in PE38 by measuring the binding of primate sera (obtained from monkeys that have been immunized with PE-based ITs) to synthetic overlapping peptides that span the sequence of PE-based RITs already began in the 1990s [32,33]. It was found that PE38 contains seven major conformational epitopes located in distinct positions on the surface of the protein: three in domain II and four in domain III [34,35]. Furthermore, it was found that these epitopes are clustered and not diffusely distributed over the entire surface. This finding facilitated the determination of the precise location of most of the epitopes by mutating bulky hydrophilic amino acids to small amino acids and demonstrating that specific mAb binding to the selected epitope was diminished or markedly reduced while cytotoxic activity was largely preserved [18,34].

One example of an RIT that was “evolved” to be more potent and less immunogenic is HA22-LR-LO10 [36]. The ancestor of this Ab is RFB4(dsFv)-PE38 (BL22), a murine anti-CD22 [37]. The first improvement was to increase its affinity to CD22 by affinity maturation, resulting in HA22-BL22 with three point mutations at the VH domain of the disulfide-stabilized Fv (dsFv) [38]. HA22 was further modified to withstand lysosomal proteases, and its immunogenicity was decreased by the removal of most of domain II of PE38 and designated HA22-LR [39]. The next modification was the identification and removal of mouse B-cell epitopes on the surface of PE38 by mutating eight bulky amino acids to small ones. This variant was named HA22-LR-8M. 

The most recent improvement was carried out in 2012 by identification and elimination of human B-cell epitopes, thereby generating HA22-LR-LO10 [36]. For this purpose, six antibody phage display libraries were constructed of scFvs isolated from six patients who developed NAbs to a PE38-containing RIT. These libraries were used to identify antibody-displaying phages that bound specifically to PE38, thereby obtaining 103 clones with unique heavy chain and light chain sequences. Competition assays showed that anti-sera from patients blocked the binding of the phage to PE38. Out of the 103 clones, 56 displayed strong binding and were used to determine the location of human B-cell epitopes on the PE38 surface [36].

The epitopes recognized were also localized to domain II of PE38. To determine which of the 56 isolated clones is specific to domain III, binding to HA22-LR was measured, identifying 41 strongly binding clones specific to domain III. These clones were then used to determine the location of the B-cell epitopes in domain III by measuring their binding to different RIT variants carrying point mutations that replaced bulky amino acids with small ones [18]. It was concluded that there are six different human B-cell epitopes, and a single mutation in each epitope, or in its vicinity, decreases the recognition of this mutant by human anti-PE anti-sera [36]. When the human epitopes were compared with the previously described mouse B-cell epitopes [18], only two overlapping positions were identified. These positions were combined with five human-specific mutations for the generation of HA22-LR-LO10 [36]. In 2013, a phase III clinical trial involving HA22 (under the name m moxetumomab pasudotox) was initiated by MedImmune.

Another RIT that was deimmunized in this manner is RG7787, a humanized Fab of the murine anti-mesothelin SS1 Ab fused to the B-cell epitope deficient toxin [40].

### 2.6. T-Cell Epitope Identification and Elimination

Another approach for reducing immune responses to RITs is to mutate potential T-cell epitopes, since T-cells are required to mount a long-lived, isotype-switched and a high affinity antibody response [41,42,43]. In 2004, Yeung et al. demonstrated that elimination of murine T-cell epitopes significantly reduced the formation of anti-interferon β NAbs in mice [44]. This led to the hypothesis that elimination of human T-cell epitopes in the bacterial portion of RITs would reduce their immunogenicity in humans, thus allowing more treatment cycles and better anti-tumor activity [45].

Initial studies in this field used computational algorithms to identify T-cell epitopes, or to narrow down the candidates [46]. T-cell epitopes are recognized by T-helper (Th) cells and are composed of peptide fragments derived from the antigen and of MHC (major histocompatibility complex) molecules [47]. Due to the variety of HLA (human leukocyte antigen) alleles in humans [48] and to the fact that T-cells stimulated by one epitope can initiate responses of numerous B-cell epitopes of the same antigen [49,50], it is a challenging task to eliminate all possible T-cell epitopes from an exogenous protein. Elimination of T-cell epitopes later became a well-accepted strategy for the deimmunization of therapeutic proteins [16,19,51,52,53,54,55].

To predict T-cell epitopes, HLA-binding algorithms are frequently used. The binding of PE38 peptides by T-cells was computed using such algorithms. The computational output was compared to the experimentally-identified T-cell epitopes, but the prediction for individual naive donors (not previously treated with PE38-containing RIT) did not correlate with the experimental data [56]. It was thus concluded that HLA class-II-binding predictions are currently not sufficient to accurately predict the T-cell epitopes in PE38, and the computational output should be supplemented by experimental data [56].

For the identification of putative T-cell epitopes, peripheral blood mononuclear cells (PBMCs) from 50 naive donors, which represent the HLA alleles’ distribution in cancer patients in the Western world, were used. The PBMCs were cultured with the intact RIT to enable its uptake by APCs (antigen-presenting cells), processing into peptides and, ultimately, the stimulation of T-cells. After the cells were expanded, T-cell epitopes were identified by re-stimulation of the specifically expanded cells with 15-mer peptides spanning the entire sequence of PE38 [57]. To detect T-cell activation, IL2-based The Enzyme-Linked ImmunoSpo (ELISpot) was used [58], identifying eight T-cell epitopes [45]. One epitope, localized to domain II, was an immunodominant and very promiscuous. As mentioned earlier, domain II can be almost entirely removed (except for the furin cleavage site) without affecting PE38 cytotoxicity (LR variants) [39]. In this study, Mazor et al. used SS1P-LR-GGS as a model, comprised of anti-mesothelin Fv fused to a truncated PE38 lacking most of domain II and containing a GGS linker between the furin cleavage site and domain III [45]. Point mutations determined by alanine scanning mutagenesis were introduced to putative epitopes in domain III. Once an alanine variant that eliminated T-cell activation was identified, it was constructed and tested for its cytotoxic activity [45].

The point mutations described above were integrated into two new RITs, LMB-T18, which targets CD22 [45] and LMB-T20, which targets mesothelin [59], each containing the same mutated toxin. When compared to its parental RIT, SS1P, LMB-T20 showed an 81% decrease in immunogenicity and, in most cases, an increased cytotoxicity in mesothelin-expressing cancer cell lines. It also induced complete xenograft regression in tumor-bearing mice [59]. LMB-T20 is thus an excellent candidate for further clinical assessment and development as a low immunogenicity RIT, as the removal of T-cell epitopes is now believed to be more effective than the removal of B-cell epitopes, since T-cells are also responsible for the downstream activation of B-cells.

During the process of identifying mutations that eliminate T-cell epitopes, two of the mutated positions were found to overlap with two of the mutated positions that eliminated B-cell epitopes: R427A and R505A [45]. Thus, T- and B-cell epitopes may overlap. Moreover, a single point mutation can eliminate both epitopes. Several reports have shown that important epitopes may be shared by B- and T-cells [60,61,62].

Finally, there are only a few published examples of non-PE-based immunotoxins that underwent deimmunization, three of which are described here. Bouganin is a type I ribosome inactivating protein (RIP) that potently blocks protein synthesis via deadenylation of rRNA. VB6-845 is an RIT comprised of deBouganin (deimmunized Bouganin) fused to a humanized Fab fragment targeting an epithelial cell adhesion molecule (EpCAM). Bouganin was deimmunized by removal of T-cell epitopes, as identified using a computational approach [16]. Efficacy studies in mice demonstrated that VB6-845 specifically and potently targeted EpCAM-positive tumors. High doses of VB6-845 administered to Cynomolgus monkeys were well-tolerated, and VB6-845 was proven to be minimally immunogenic in these monkeys [63].

The second example is the deimmunization of a fragment of diphtheria toxin (DT) by elimination of B-cell epitopes. This deimmunization was based on point mutations that were introduced to replace surface-exposed R, K, D, E and Q amino acids, all distal to the catalytic site (based on the known 3D structure of DT) of a truncated diphtheria-toxin (DT390). Upon immunization with the mutants using mice of two different MHC haplotypes, the level of anti-toxin antibodies was reduced by 90%, compared to mice that were challenged with the wild-type toxin [64].

The third example is the small (17 kDa) fungal ribotoxin α-sarcin. Two T-cell epitopes of the toxin were identified using peptide mapping. Several mutations within these putative epitopes were tested individually and in combination with deimmunized α-sarcin variants, showing the desired properties of silencing T-cell epitopes and retention of potency. A deimmunized variant (only two point mutations, D9T/Q142T, were required to deimmunize α-sarcin) demonstrated a complete lack of T-cell activation in vitro, as revealed by stimulating peripheral blood mononuclear cells from human donors with diverse HLA allotypes. Finally, an immunotoxin comprised of a fusion of the deimmunized D9T/Q142T α-sarcin variant to a scFv targeting Her2 demonstrated potent cell killing, equivalent to a fusion protein comprising the WT α-sarcin [65].

## 3. Immunocytokines

Cytokines are a group of small immunomodulatory proteins that either activate or inhibit the immune system in health and disease. The activation or inhibition depend on the cytokines properties and concentration, their receptors and environment [66,67]. Cytokines are mainly secreted by leukocytes, but also by fibroblasts, endothelial cells and other stromal cells. Usually, cytokines act locally (autocrine or paracrine) by binding with high affinity to their receptors and regulating the activity of immune cells [68]. Cytokines can act on distant organs in some pathological conditions, such as cancer, affecting biological processes, e.g., control of body temperature, vascular permeability and leukocyte development [68].

Several pro-inflammatory cytokines, including interleukin-2 (IL2), tumor necrosis factor (TNF) and interferon-α (IFNα), have been approved for clinical use in certain types of cancer [67]. The systemic administration of recombinant cytokines as therapeutic agents might face some obstacles, such as wide expression of cytokine receptor on many types of cells and tissues, which can cause subsequent off-target toxicity, especially for potent pro-inflammatory cytokines. Furthermore, the inability to reach an effective concentration at the site of disease could lessen pharmaceutical activity. Thus, fusing cytokines to antibody fragments that specifically bind tumor-associated antigens could allow selective localization to the tumor or disease site and should increase the therapeutic index of the cytokine payload [69,70]. Most of the studies of immunocytokines discussed here were carried out by the group of Dario Neri of the Swiss Federal Institute of Technology, ETH, Zurich, Switzerland. Antibodies can be used as full IgGs or as fragments (Figure 2), each showing different advantages. Full IgG has a longer serum half-life, whereas smaller fragments, such as scFv and Fab, penetrate the tumor more easily and dissipate evenly inside the cell [66,71]. The first experimental application of immunocytokines relied on the full IgG format, but in subsequent studies, smaller antibody fragments, mainly scFv and diabodies, were preferred [67], since antibody fragment-based immunocytokines, composed of only two protein moieties, were easier to manufacture and presented a favorable tumor:organ ratio in mouse cancer models [67,72,73,74]. Targets that were evaluated for antibody-targeted cytokines include extracellular matrix proteins, such as alternatively-spliced isoforms of fibronectin (EDA and EDB) and tenascin C [75,76], or cell surface proteins, such as CD64 and CD30 [77,78].

Unlike immunotoxins and other antibody-targeted human effector proteins, such as immunoRNAses and targeted granzymes (see Section 4.5 below), immunocytokines do not internalize into cells and are not cytotoxic on their own. Rather, immunocytokines are able to activate various components of the immune system to act against neoplastic cells [67]. Changes in cytokine concentrations at the target-tumor site may elevate the pre-existing immune response towards neoplasms [79,80,81]. Immunocytokines can bridge tumor cells and leukocytes, such as T-cells or NK cells [82]. Increased density of leukocytes at neoplastic mass was demonstrated following pro-inflammatory immunocytokine treatment both in mouse models and in cancer patients [83,84,85]. In addition, immunocytokines can activate leukocytes selectively at the site of tumor or disease. The type of leukocytes activated is determined by the type of cytokine payload [83,86].

The cytokines most commonly used for fusion are IL2, TNF and the heterodimeric IL12, all pro-inflammatory [70,87,88]. There are currently 10 immunocytokine molecules under clinical trials for cancer treatment (Table 1). These molecules consist of the aforementioned cytokines and an antibody moiety in the form of an IgG, scFv or a diabody [89,90,91,92,93]. These immunocytokines can be administered as a stand-alone therapy or in combination with other therapeutic agents, such as drugs (e.g., doxorubicin, paclitaxel, usually resulting in a synergistic effect) [79,94], mAbs (e.g., rituximab) [81], radiotherapy [90] and other immunocytokines [84,86,95]. The combination of two immunocytokines may result in steric hindrance, which eliminates the targeting properties of the two parental immunocytokines [86].

### 3.1. IL2-Based Immunocytokines

NHS-IL2, also known as “selectikine” is an immunocytokine consisting of human NHS76, an IgG antibody specific for necrotic DNA, fused to genetically-modified human IL-2 that selectively activates the high-affinity IL-2 receptor. When tested in a mouse model with syngeneic Lewis lung carcinoma, NHS-IL2 alone did not exhibit any significant effect on tumor growth, but the combination with cisplatin (a chemotherapy drug) and radiotherapy resulted in marked tumor size reduction. On Day 53, five out of six mice receiving the combined therapy exhibited complete tumor regression [90]. When tested in a clinical trial on human patients with stage IV non-small cell lung cancer (carcinoma), several adverse effects (AEs) were observed, mainly fatigue, anorexia and rash, all followed by complete recovery within the second week of the treatment cycle. Overall, only three Grade 3 AEs (severe AEs) were directly related to NHS-IL2, namely normochromic normocytic anemia, lymphopenia and malignant pericarditis [90]. In addition to the mild transient rash, the only classic IL2 immune-related AE was thyroiditis, which all patients eventually developed during treatment [90]. Two patients were long-term survivors with no signs of active disease nearly a year later. 

L19-TNF is an armed antibody that selectively targets human TNF to EDB of fibronectin on tumor blood vessels. L19-TNF is currently under phase I/II clinical trials in patients with various solid tumors. Thirty-four patients with advanced solid tumors were treated with L19-TNF in the Day 1-3-5 treatment regime for a three-week cycle. Patients were divided into two groups: 22 patients in phase I (sub-divided into six cohorts) and 12 patients in phase II. Repeated intravenous administration was safe up to 13 μg/kg (the maximum dose used in the trial). In general, L19-TNF-associated toxicity was mild and transient, mostly including constitutional symptoms, such as chills, fever, fatigue and pain, and was not dose dependent. During phase I, AEs were mostly L19-TNF-related (387/507). Seven severe AEs occurred in five patients in phase I, of which only one was L19-TNF-related. During phase II, 142/235 AEs were treatment-related, and none of the eight severe AEs were L19-TNF-related [96].

Tumor response was evaluated in 21 patients from the phase I group and 10 patients from the phase II group. Stable disease (SD) was attained in 66.67% of phase I patients and in 50% of phase II patients. For phase I patients with SD, median time-to-progression (TTP) was 134 days, and median progression-free-survival (PFS) was 84 days. TTP and PFS for phase II patients were 85 and 77 days, respectively. Median overall survival for was 226 days (*n* = 17) and 110 days (*n* = 10) for phase I and phase II patients, respectively [96].

Hu14.18-IL2, also known as “APN301” is a fusion protein consisting of IL2 linked to a humanized monoclonal antibody that recognizes the GD2 disialoganglioside expressed on neuroblastoma cells. Hu14.18-IL2 was evaluated in a phase II study in two strata of patients with recurrent or refractory neuroblastoma. It was concluded that patients with lower tumor burden (as evaluated by metaiodobenzylguanidine (MIBG) and/or bone marrow histology) had a 21.7% complete response (CR) rate upon treatment with hu14.18-IL2, whereas patients with bulky disease did not respond. The authors suggested that Hu14.18-IL2 should be further tested in children with non-bulky high-risk neuroblastoma [91].

### 3.2. IL4- and IL10-Based Immunocytokines

Immunocytokines may also be used to treat ailments other than cancer, such as inflammation and autoimmune diseases [66]. In such cases, the anti-inflammatory cytokines IL4 and IL10 are preferable. DEKAVIL (F8-IL10), a clinical-stage immunocytokine, is targeted to EDB of fibronectin and inhibits the progression of collagen-induced arthritis. Administration of IL10-based immunocytokines to a mouse arthritis model significantly reduced the arthritic score compared to the control [97]. A phase IB clinical trial carried out in 2014 demonstrated that administration of DEKAVIL to rheumatoid arthritis patients, who previously failed at least one line of anti-TNF treatment, in doses up to 300 µg/kg, combined with a fixed dose of methotrexate, did not reach the maximum tolerated dose. No dose-limiting toxicity and serious or unexpected AEs were observed, with all reported AEs resolved after treatment completion, following little or no therapeutic intervention. Beneficial effects of treatment were observed in all patients even at low drug concentrations, starting at the initial steps of dose escalation [98]. In addition, IgG-based IL10 fusion proteins for treating inflammatory disorders and autoimmune disease are described in a Roche-Glycart patent [99].

## 4. Addressing the Immunogenicity of the Targeting Antibodies

The potential or observed immunogenicity of targeted protein therapeutics was identified shortly after bacterial or plant toxins were first used to generate immunotoxins. It was also found that most of the immunogenic epitopes were of the non-human protein cargo. Accordingly, efforts were invested in reducing the cargo immunogenicity, as described in the first section of this article, or in developing antibody-targeted human effector proteins as described in this articles and in other articles of this special issue. In parallel, motivated by the requirement to reduce their immunogenicity, the field of therapeutic antibodies started replacing murine monoclonal antibodies with chimeric (first reported in [100]), humanized (first reported in [101]) and finally fully-human antibodies. Chimeric antibodies are about 70% human and possess a human Fc that fully allows them to interact with human effector cells and the complement cascade [102]. Technical advances in the antibody engineering field made it possible to further reduce the murine part of mAbs by replacing only the complementarity-determining region (CDR) loops of a fully-human antibody with the CDR loops of the parental murine mAb, an approach initially called “reshaping” and later known as complementarity-determining region grafting [101,103]. Such “humanized” antibodies are 85–90% human and as such are expected to be even less immunogenic than chimeric antibodies. The generation of fully-human recombinant antibodies was initially made possible by phage display technology [104,105] and later by developing transgenic mice carrying parts of the human antibody loci and producing human antibodies [106,107]. The fields of targeted protein therapeutics and antibody-drug conjugates (ADCs) also implemented the replacement of murine monoclonal antibodies, as shown in the examples presented below.

### 4.1. Antibodies That Target Immunotoxins

Immunotoxins including the effector molecule PE were initially developed as chemical conjugates linking mouse mABs to the toxin [108,109]. Immunotoxins where the effector molecule was derived from the plant toxin ricin or from the bacterial DT were also initially prepared by chemical conjugation of the toxins to polyclonal or monoclonal antibodies of non-human origin [110,111,112].

The PE-based first generation antibody-toxin conjugates were soon replaced by recombinant single-chain immunotoxins, where a scFv coding gene (originally cloned from a murine hybridoma) was fused to parts of the toxin-coding gene [5,113]. Later on, as chimeric, humanized and human antibodies became more common, so was their selection as targeting moieties for targeted protein therapeutics and for ADCs. An early example is the replacement of the murine anti-IL2 receptor antibody, anti-Tac, which was used in a first-generation immunotoxin [109], with the humanized version of anti-Tac as a stand-alone therapeutic IgG in a second-generation single-chain immunotoxin [114,115]. 

The first published example for humanizing the antibody component of an immunotoxin was the humanization of the anti-Lewis^Y^ scFv B3 derived from a mouse monoclonal antibody by a process called “framework exchange” [13]. The authors showed that humanized B3(Fv)-PE38 lost immunogenic epitopes recognized by sera from monkeys that had been immunized with B3(Fv)-PE38, thus potentially increasing the efficacy of B3 and B3 immunotoxins in cancer therapy and diagnosis, which may be limited by the human anti-mouse response. Nonetheless, the non-human nature of PE is responsible for most of the anti-immunotoxin immune responses observed in non-human primates and in human patients upon treatment.

The human or non-human nature of the antibody can be roughly ascribed to the year its development was initiated. Molecules whose development began before the mid-1990s usually have a murine antibody, while later on, molecules were generated with a chimeric, humanized or human antibody. For example, an anti-CD33 immunotoxin based on an already humanized antibody, named Hum195 [116,117], conjugated to the ribosome-inactivating protein gelonin was reported in 1994 [118]. The group of Stefan Barth had been studying anti-CD64 immunotoxins since the 1990s. CD64, a high affinity receptor for IgG (FcγRI), is expressed on acute myeloid leukemia blast cells. They were using PE as the effector molecule. Initially, they used a murine anti-CD68 antibody, m22, for targeting CD64 [119]. Later, this group switched to a humanized anti-CD64 antibody, HA22 scFv, for targeting their immunotoxins [120]. A recent example of humanizing antibodies to target immunotoxins is from the group of Mitchell Ho at NIH. This group is studying antibodies that target glypican-3 (GPC3), a cell-surface heparan sulfate proteoglycan highly expressed in hepatocellular carcinoma (HCC). Initially, this group generated a set of high-affinity mABs targeting GPC3 and, after cloning the corresponding scFvs, tested them as PE-based immunotoxins. Later, they humanized two of these antibodies using an approach they called “dual CDR grafting”, showing that the humanized antibodies, expressed as IgGs, could inhibit HCC tumor xenograft growth in nude mice by ADCC and CDC [121].

### 4.2. Antibodies That Target Radioimmunoconjugates

Radioimmunoconjugates (RICs) and antibody-drug conjugates (ADCs) are forms of armed antibodies in which, instead of a protein, the cargo is a small chemical entity and is therefore hardly ever immunogenic by itself. RICs and ADCs are not covered in this Special Issue, but nonetheless, we will provide a few examples of using chimeric, humanized or human antibodies for targeting such drugs.

There are currently only two Food and drug administration (FDA)-approved RICs, Bexaar (^131^I-tositumomab) and Zevalin (^90^Y-ibritumomab tiuxetan), both targeted by anti-CD20 mouse mABs and used to treat non-Hodgkin lymphoma (NHL) [122]. HAMA response is usually diminished in NHL patients following chemotherapy. In chemotherapy-naive patients, the HAMA response can be considerable, as was found for Bexaar [122,123]. RICs were later developed based on humanized antibodies. One such RIC is ^90^Y-epratuzumab (hLL2), an anti-CD22 agent that was initially developed using the murine mAB LL2 [124], which was subsequently humanized [122]. More than 15 years later, ^90^Y-epratuzumab is still undergoing clinical evaluation [125]. An additional RIC, developed by David Goldenberg’s group at Garden State Cancer Center in New Jersey, is based on the PAM4 IgG, a mouse mAB that recognizes a unique epitope associated with a mucin found almost exclusively in pancreatic cancer. Recently, this group reported a new pre-targeting procedure for delivering therapeutic radionuclides using ^90^Y-humanized PAM4 IgG (^90^Y-clivatuzumab tetraxetan) [126]. A final example is ^131^I-bevacizumab, a RIC-based on the FDA-approved anti-angiogenic therapeutic mAb bevacizumab (Avastin), which was labeled with ^131^I and shown to inhibit the growth of VEGF-overexpressing cultured ovarian cancer cells and xenografts [127].

### 4.3. Antibodies That Target Antibody-Drug Conjugates

The antibody-drug conjugates (ADCs) that have been developed since the 1980s followed a similar trend, as the more veteran agents (that did not gain regulatory approval) were based on mouse mABs, while later-developed ADCs were based on non-murine antibodies [128,129]. The first FDA-approved ADC, Mylotarg (gemtuzumab ozogamicin), was initially licensed in 2000 for relapsed acute myeloid leukemia using a humanized IgG4 anti-CD33 antibody and was later withdrawn from the market. The more recently approved ADC brentuximab vedotin (Adcetris) was based on a chimeric antibody, the anti-CD30 cAC antibody [130]. Adcetris was FDA-approved in 2015 for “Hodgkin lymphoma patients who fail autologous hematopoietic transplantation or who fail at least two prior multi-agent chemotherapy regimens and are not autologous hematopoietic transplantation candidates”. An additional example of “repurposing” an FDA-approved therapeutic mAb for drug delivery is the case of trastuzumab emtansine, an anti-ErbB2 agent approved in 2003 for treating breast cancer patients [131].

In the final section of this review article, we will refer to antibodies that are used to target human effector proteins, the main theme of this Special Issue. Since antibody-human effector protein fusions are relatively “young” compared to immunotoxins and ADCs, they are rarely targeted by a mouse antibody.

### 4.4. Antibodies That Target Immunocytokines

Several immunocytokines have so far been developed. For example, a human antibody is used to target NHS-IL2, an immunocytokine consisting of an antibody specific for necrotic DNA fused to genetically-modified human interleukin IL-2, which selectively activates the high-affinity IL-2 receptor [90]. A human mAB is used to target L19-TNF, an armed antibody that selectively targets human TNF to EDB of fibronectin on tumor blood vessels [96]. A human mAB, F8, is used to target DEKAVIL (F8-IL10), a clinical-stage immunocytokine targeted to EDB of fibronectin (EDB), thereby inhibiting the progression of collagen-induced arthritis [97]. A humanized antibody is used to target hu14.18-IL2, a fusion protein consisting of IL2 linked to an antibody that recognizes the GD2 disialoganglioside expressed on neuroblastoma cells [91]. A number of preclinical immunocytokines, which include the cytokines IL10, IL2, IL4 and IL12, are targeted by human antibody fragments [66].

### 4.5. Antibodies That Target Granzymes and ImmunoRNAses

Granzymes and ImmunoRNAses are discussed in other articles of this Special Issue. Similar to the immunocytokines described in the previous section, they are also “young” compared to immunotoxins and ADCs, and therefore, most (but not all) are targeted by non-murine antibodies. We will give a few examples of antibodies that were applied to target such molecules.

#### 4.5.1. Targeted Granzymes

Granzymes are serine proteases that are released by cytoplasmic granules within cytotoxic T-cells and natural killer (NK) cells. Gm-H22(scFv) is a fusion protein comprised of granzyme M fused to a humanized scFv (H22), which specifically binds to CD64. This humanized cytolytic fusion protein specifically targeted the acute myeloid leukemia cell line HL60 and patient-derived primary leukemic cells [78]. GrB/4D5 is a fusion protein comprised of granzyme B fused to the scFv of the FDA-approved, humanized therapeutic mAb Herceptin. GrB/4D5 was shown to inhibit the growth Her2/neu-positive tumor cells [132].

Some granzymes are still targeted by murine antibodies. GrB/scFvMEL is a GrB fusion protein composed of human granzyme B and the single-chain antibody scFvMEL. This scFv was cloned from a mouse mAB, which targets the melanoma gp240 antigen and exerts extensive cytotoxic effects by inducing apoptosis of treated melanoma cells, as well as growth inhibition of human melanoma xenografts in nude mice [133,134]. 

A scFv derived from the anti-ErbB2 mouse monoclonal antibody was used to target Grb. This molecule, called GrBscFv(FRP5), was shown to inhibit the growth of cultured breast cancer cell lines via an apoptotic mechanism [135].

#### 4.5.2. ImmunoRNAses

ImmunoRNAses are RNases that are linked to targeting antibodies or ligands. The immunoRNAse ranpirnase (based on a frog RNase) is targeted with a humanized, internalizing, anti–Trop-2 antibody, which recognizes a cell surface glycoprotein overexpressed in a variety of epithelial cancers. Ranpirnase was shown to induce potent cytotoxicity against diverse epithelial cancer cells [136]. More recently, a humanized anti human EGF receptor(-EGFR) ranpirnase-diabody fusion protein with the specificity of cetuximab (targeting the EGF receptor) was shown to deliver two ranpirnase moieties per molecule to EGFR-positive tumor cells, resulting in a superior cytotoxic effect compared to the corresponding monovalent counterpart [137]. The same group also described an anti-CD22 conjugate between ranpirnase and an IgG4-reformatted humanized version of the monoclonal antibody RFB4, achieving significant in vitro cytotoxicity towards lymphoma and leukemia cell lines [138]. Polyvalent recombinant humanized anti-Trop-2 antibodies targeting tetrakis-ranpirnase with potent antitumor activity against breast cancer were recently described by a group from the company Immunomedics [139]. 

As for antibody-targeted human RNAses, the preparation and characterization of hERB-hRNAse, a fully-human antitumor immunoRNAse, was reported in 2004. It was constructed as a fusion between a human RNase and an anti-ErbB2 human scFv, shown to be a potent killer of ErbB2-expressing cells in vitro and to inhibit the growth of human tumor xenografts in nude mice [140]. An anti-CD30 immunoRNAse, Ber-H2-scFv-hpRNase, combining human pancreatic ribonuclease with a murine scFv, was described in 2007. The antibody used to target Ber-H2-scFv-hpRNase was BerH2, a scFv cloned from anti-CD30 hybridoma, Ber-H2-scFv-hpRNase revealed CD30-specific anti-tumor activity in BALB/c mice following challenge with CD30-positive tumor cells [141]. One year later, the authors reported an anti-CD30 immunoRNAse developed for CD30^+^ lymphomas that was targeted by a human anti-CD30 antibody. The CD30-specific human scFv, 4E3, was obtained from a semisynthetic human antibody library using phage display and was converted into the scFv-Fc format by fusion with the human IgG1-Fc part, as well as into the human IgG1 format. Several antibody-human pancreatic RNAse fusion formats were evaluated in the course of that study, including scFv fusion, scFv-RNAse-Fc fusion and scFv-Fc-RNAse fusion. The authors reported that the scFv-Fc-RNase format was most favorable for the construction of an entirely human CD30-specific immunoRNAse [77]. Finally, in 2014, as a study of a therapeutic antibody development platform, several fusion proteins consisting of tumor-specific human IgGs and human pancreatic RNases were evaluated. Human antibodies specific to several tumor targets were used in that study, including anti-mesothelin and MN-antigen (a tumor associated antigen carbonic anhydrase IX, CA9) [142].

## 5. Summary

The immunogenicity of recombinant ITs is a critical issue for their repeated use [9]. Third generation ITs consist of a human or humanized targeting moiety and a foreign cytotoxic domain, mostly of bacterial or plant origin. The latter makes IT molecules immunogenic, thus preventing repeated treatment cycles. To overcome the immunogenicity of the cytotoxic component, several approaches have been implemented, including the removal of epitopes recognized by B- and T-cells, combined treatment with immunosuppressive agents and using human cytotoxic fusion proteins.

In the first section of this article, we reviewed the long and arduous deimmunization process that PE-based immunotoxins underwent, with a focus on the immunodominant toxin component derived from a bacterial origin. Following unsuccessful attempts to reduce the immunogenicity by PEGylation, B-cell epitopes were identified and removed, as well as parts of the toxin found to be unnecessary for some applications, and T-cell epitopes were identified and removed. Present-day immunotoxins that combine these modifications have a toxin component almost half the size that was used in the late 1980s (PE40 was used then; PE24 is used now, with the number representing the molecular weight of the toxin component). Currently-used deimmunized immunotoxins carry many point mutations, but still preserve potency [31].

In the second section of this article, we reviewed immunocytokines. Cytokines are small proteins that regulate the activity of the immune system, depending on the type of cytokine, its concentration and the environment in which it is active. The targeting moiety of immunocytokines directs them specifically to the tumor or disease site, where the cytokine moiety can selectively activate leukocytes to act against cancerous cells or inflamed tissue. Currently, there are approximately 10 immunocytokines undergoing different phases of clinical trials. Further research is required to determine which cytokine is preferable for each cancer indication and which pharmaceutical agents could benefit most from combination treatment with immunocytokines [67].

In the third section of this article, we addressed the immunogenicity of the targeting antibody component of antibody-targeted drugs. We provided examples for antibodies used as the targeting component of immunotoxins, RICs, ADCs, immunocytokines, antibody-targeted granzymes and immunoRNAses. The trend that emerges is that molecules whose development began before the 1990s were targeted by a mouse antibody. Most molecules whose development began later are targeted by a humanized or human, rather than a mouse, antibody. Many features of targeted drugs may contribute to limiting the anti-drug immune response [143]. We can conclude that, although the antibody part, and in particular a small antibody fragment such as a scFv or a diabody, plays a minor role in the immunogenicity of an antibody-targeted protein drug, the following long-term exposure of patients, as many therapeutic regimes require, may become a significant challenge; thus ideally, this should be kept to the absolute possible minimum.

## Figures and Tables

**Table 1:** List of anti-cancer immunocytokines currently under clinical trials.

Immunocytokine Name	Cytokine Used	Target Antigen	Format
Hu14.18-IL2	IL2	GD2	IgG
NHS-IL2		Nuclear antigen	
Anti-CEA-IL2v		CEA	
DI-Leu16-IL2		CD20	
HuKS-IL2		EpCAM	
NHS-IL12	IL12	Nuclear antigen	
BC1-IL12		Domain 7 of FN	
L19-IL2	IL2	EDB of FN	Diabody
F16-IL2		A1 of Tn-C	
L19-TNF	TNF	EDB of FN	scFv

GD2, disialoganglioside; nuclear antigen, necrotic DNA; CEA, carcinoembryonic antigen; EpCAM, epithelial cell adhesion molecule; Domain 7 of FN, epitope on fibronectin domain 7, which is hidden in the presence of EDB; EDB of FN, alternatively spliced extra domain B of fibronectin; A1 of Tn-C, alternatively spliced A1 domain of tenascin-C.
